# Review of Applications and Future Prospects of Stimuli-Responsive Hydrogel Based on Thermo-Responsive Biopolymers in Drug Delivery Systems

**DOI:** 10.3390/polym13132086

**Published:** 2021-06-24

**Authors:** Sudipta Chatterjee, Patrick Chi-leung Hui

**Affiliations:** Institute of Textiles and Clothing, The Hong Kong Polytechnic University, Hung Hom, Hong Kong; sudipta.chatterjee@polyu.edu.hk

**Keywords:** thermo-responsive hydrogel, biopolymer, drug delivery, LCST, polysaccharide, transdermal therapy

## Abstract

Some of thermo-responsive polysaccharides, namely, cellulose, xyloglucan, and chitosan, and protein-like gelatin or elastin-like polypeptides can exhibit temperature dependent sol–gel transitions. Due to their biodegradability, biocompatibility, and non-toxicity, such biomaterials are becoming popular for drug delivery and tissue engineering applications. This paper aims to review the properties of sol–gel transition, mechanical strength, drug release (bioavailability of drugs), and cytotoxicity of stimuli-responsive hydrogel made of thermo-responsive biopolymers in drug delivery systems. One of the major applications of such thermos-responsive biopolymers is on textile-based transdermal therapy where the formulation, mechanical, and drug release properties and the cytotoxicity of thermo-responsive hydrogel in drug delivery systems of traditional Chinese medicine have been fully reviewed. Textile-based transdermal therapy, a non-invasive method to treat skin-related disease, can overcome the poor bioavailability of drugs from conventional non-invasive administration. This study also discusses the future prospects of stimuli-responsive hydrogels made of thermo-responsive biopolymers for non-invasive treatment of skin-related disease via textile-based transdermal therapy.

## 1. Introduction

**Figure 1:**
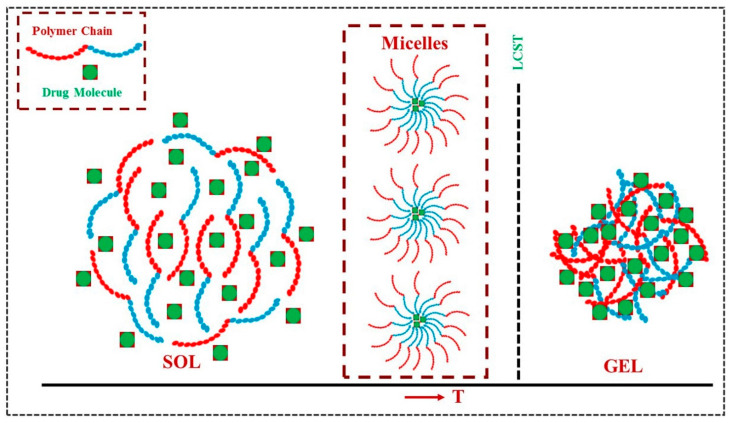
The sol–gel transition of LCST-type thermo-responsive polymer-based drug delivery system (Schematic presentation). Formulation over LCST changes to hydrogel from the solution state. LCST-type thermo-responsive polymer in solution forms micelles at low concentration and that further aggregates at high polymer concentration to form gel at a temperature (≥LCST).

**Figure 2:**
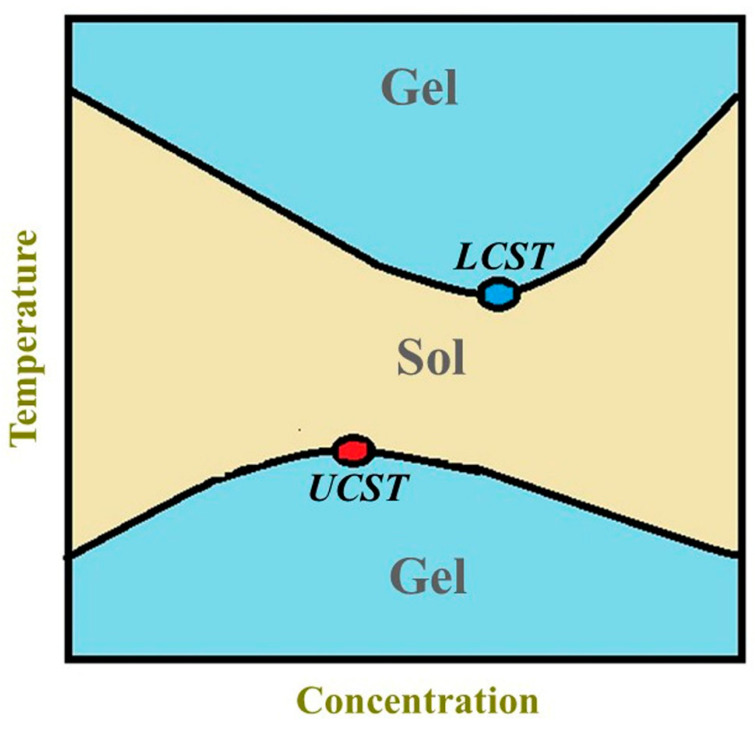
LCST-type formulation undergoes sol–gel transition with increase in temperature while UCST-type formulation undergoes sol–gel transition as the temperature decreases. The black colored curved lines indicate the boundary of phase separation.

Hydrogels are widely being applied in biomedical areas for drug delivery because of their advanced properties such as biocompatibility, biodegradability, and nontoxicity [1]. The high water content in hydrogels and physically or chemically crosslinked polymeric network render control over their physicochemical properties and spatiotemporal control over the release of various drugs and therapeutic agents [2]. Hydrogels are applied for wound dressings, tissue engineering, bio-sensing, bio-printing, and electrospinning [3]. Attention to stimuli-responsive hydrogels has been growing in recent years as they are capable of being modulated under the influence of external stimuli including temperature, pH, light, and ultrasound [4]. The stimuli-responsive hydrogels are considered as smart drug delivery systems that are capable of providing spatiotemporal control over drug release and can effectively protect labile drugs from degradation [5,6,7,8]. The thermo-responsive polymers with lower critical solution temperature (LCST) are promising candidates for biomedical applications as these polymers only form hydrogels above its LCST [9,10]. The thermo-responsive hydrogel formation with sol–gel transition at LCST is schematically given in Figure 1. The sol–gel transition of a thermo-responsive polymer at LCST thermodynamically resembles the phenomenon of temperature-induced folding of a protein [4]. The polymers tend to aggregate with rise in temperature imparting the positive ΔS and negative free energy (ΔG) of aggregation, and the hydrophobic effect becomes the guiding force for gel formation at LCST. In the case of thermo-responsive polymers with an upper critical solution temperature (UCST), only one liquid phase will remain above the UCST but upon cooling, separation into two phases occurs when the temperature given by the equilibrium curve is reached. UCST-polymers show strong supramolecular polymer–polymer interactions, and UCST behavior is enthalpy driven. The phase separations of LSCT- and UCST-type formulations are given in Figure 2, and the phase separation boundary corresponds to the cloud point of a solution. As in the case of an LCST-polymer at temperatures higher than the cloud point, at temperatures lower than cloud point of an UCST polymer, the Gibbs energy of dissolution turns positive [11]. Thermo-responsive polymers, which are capable of showing sol–gel transition at 37 °C, are more suitable for drug delivery applications as these polymers can show in situ hydrogel formation and allow the encapsulation of drug and therapeutics at body temperature conditions [12]. The thermo-responsive polymers with in situ gel formation can fill wounded cavities by taking their shape, and the local injection of the polymer solution by syringe, which is minimally invasive, can effectively minimize systemic toxicity of chemotherapy drugs alongside improvement of patient compliance [13]. Therefore, thermo-responsive gel systems can serve as a depot after in situ gel formation, and it shows controlled and timely release of drugs inside the body mitigating the need for multiple daily dosing [14].

The drug delivery systems formed by hydrogels of synthetic thermo-responsive polymer poloxamers (Pluronics^®^) are of great interest especially for transdermal, injectable, ophthalmic, and vaginal administration of drug and therapeutics [15,16]. Poly(*N*-isopropylacrylamide) (pNIPAm) is also an intensely studied synthetic thermo-responsive polymer, and this fascinating polymer offers multi-directional biomedical applications as its LCST is very close to body temperature and capable of showing fast on off switching [17,18]. The hydrogels developed from pNIPAm and poly(N-butylacrylamide) (50:50) showed a sustained release of antimitotic colchicine to human vascular cells over a considerable period of time and did not show any cell toxicity as evidenced by cell viability test [19]. A nanofibrous thermal-responsive drug delivery system was developed by co-axial electrospinning technique cross-linked using copolymer poly(*N*-isopropylacrylamide-*co*-*N*-isopropylmethacrylamide) and the system showed self-regulated drug release properties [20]. A series of pNIPAm-based thermo-responsive biodegradable hydrogels were developed using NIPAm and two biodegradable cross-linkers, poly(ε-caprolactone) dimethacrylate and bisacryloylcystamine, and levofloxacin-loaded hydrogels exhibited a thermo-induced, slow sustained drug release and a reduction-induced fast release [21]. A pNIPAm-based biomimetic multifunctional nanostructured pillow with fast photo-thermal responsiveness for near-infrared, light-controlled, on-demand drug delivery was developed that showed a high biocompatibility and a controlled release of drug molecules into pig tissue [22]. The thermo-responsive injectable hydrogel made from graft copolymers of alginate backbone and *N*-isopropylacrylamide/*N*-*tert*-butylacrylamide random copolymer showed sol–gel transition near body temperature and the excellent shear-induced injectability of the formulation at room temperature made it suitable for potential cell transplantation applications [23].

Poloxamers are water-soluble, non-ionic triblock copolymers of poly(ethylene oxide)-*b*-poly(propylene oxide)-*b*-poly(ethylene oxide) and form viscous hydrogels at body temperature through the supramolecular interactions of their polymeric blocks. Poloxamers are amphiphilic molecules that maintain balance between the hydrophobic and hydrophilic properties of their different segments, and that is responsible for the solvation in an aqueous solution at low temperatures via hydrogen bonding interactions between water and polymers. With the rise in temperature, the random motion of copolymers is increased, and the association of copolymers’ hydrophobic parts leads to micelle formation. The gel state is attained at the critical gelation concentration (CGC) of polymer where the micelles are packed closely enough to form the gel. The mechanism of gelation in Pluronics systems was proposed by Alexandridis et al. [24] where the gelation was driven by the reduced polarity of ethylene oxide and propylene oxide segments upon rise in temperature and a gain in entropy upon the aggregation of the polymers. Poloxamer P407 or Pluronic F-127 (PF127) is the most studied thermo-responsive polymer of Pluronics^®^ and has received special attention in bio-medical and pharmaceutical areas, especially for drug delivery and tissue regeneration based on rheological properties, biocompatibility, and low cytotoxicity [15,25]. Among several advantages of PF127-based formulations, the toxicity of PF127-based drug delivery systems on cells was reported by several research works [26,27,28]. The PF127-based dual-responsive hydrogels (with/without) loaded Cortex Moutan showed low cytotoxicity on human HaCaT keratinocytes, and the cell viability was just above 80.0% within the concentration range of 0.0–20.0 μg/mL [28]. The PF127-based hydrogel with loaded Cortex Moutan (Traditional Chinese medicine) did not show cytotoxicity on human HaCaT keratinocytes within the concentration range of 0.0–20.0 μg/mL, but these hydrogel formulations were found to be slightly cytotoxic without being drug-loaded [29]. The PF127-based micelles exhibited low cytotoxicity on the Bel 7402 and L02 cells, and the cell viability was just above 85.0% within the concentration range of 12.5–200.0 μg/mL [27]. The gene delivery vectors developed from PF127-poly (dimethylaminoethyl methacrylate) and PF127-poly (dimethylaminoethyl methacrylate-tert-butyl acrylate) showed high cytotoxicity on human embryonic kidney 293T cell line. The cell viabilities were 50.0% and 40.0%, respectively, at a concentration of 12.5 μg/mL, while the cell viability was reported to be ~90.0% with PF127-poly (dimethylaminoethyl methacrylate-acrylic acid) at the same concentration [26].

**Figure 3:**
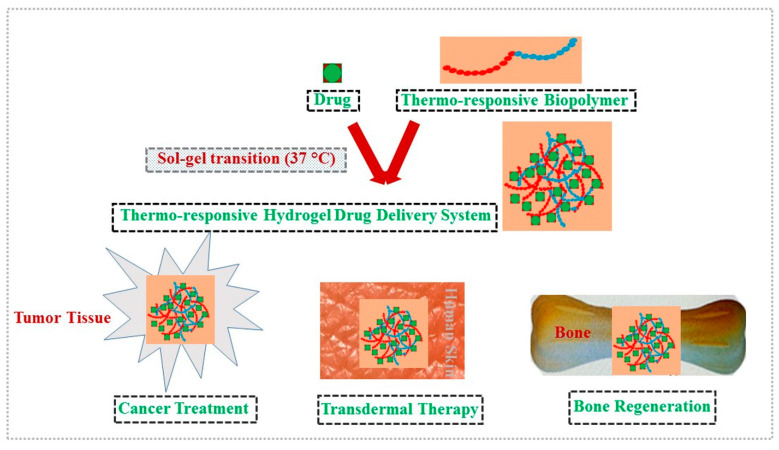
The formation of drug-loaded biopolymer-based thermo-responsive hydrogel system via in situ gel formation and its bio-medical applications including cancer treatment, transdermal, and bone regeneration (Flow-chart presentation).

This review encompasses hydrogels made of bio-polymers which are capable of showing thermo-responsive properties with desired rheological properties, and have biocompatibility, biodegradability, and no cytotoxicity. The schematic representations of in situ hydrogel formation by such bio-polymers and their biomedical applications are given in Figure 3. Furthermore, the polymers with biological origin have several advantages over synthetic polymers including metabolic removal of byproducts and enzymatic degradation [30]. The formulations of thermo-responsive biopolymers are subject to physical mixing with other biomaterials to improve their drug delivery and tissue engineering applications [30]. The novelty of this review is to analyze thermo-responsive polymers in their drug delivery applications, and the future prospects of these bio-based formulations as transdermal drug delivery systems are discussed.

## 2. Thermo-Responsive Polysaccharides and Their Drug Delivery Applications

**Figure 4:**
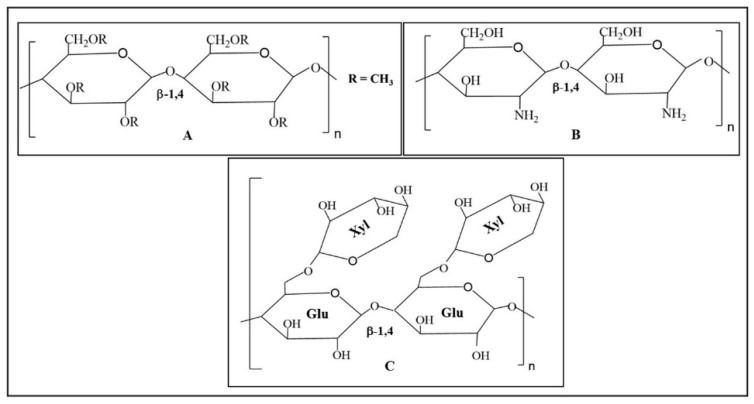
The chemical structures of thermo-responsive polysaccharides: (**A**) methylcellulose (water-soluble derivative of cellulose); (**B**) chitosan; and (**C**) xyloglucan. The chemical structures of the compounds are drawn using ChemDraw Prime software.

The thermo-responsive polysaccharides has been of great interest in biomedical areas, especially for drug delivery applications due to their biodegradability, non-toxicity, inherent biocompatibility, wide availability, and embracing functionality [31]. The thermo-responsive polysaccharides, which show upper critical solution temperatures (UCST) including carrageenans, starch, agarose, and gellan gum, are rarely accepted for drug delivery applications as the solution state is attained at a temperature that is much higher than the physiological temperature [4]. Over the last few years, it has been a growing interest in developing drug delivery systems of thermo-responsive polysaccharides which exhibit LCST [6,9]. The chemical structures of some useful thermo-responsive polysaccharides are given in Figure 4.

Cellulose, a linear polysaccharide consisting of *β* (beta)-1,4 linked glucose units, is the most abundant biopolymer in nature. Methylcellulose is chemically derived from cellulose and the hydroxyl functional groups (-OH) of cellulose are substituted by the methoxy group (-OCH_3_) in methylcellulose (Figure 3). Methylcellulose is a water-soluble, thermo-responsive polymer and can exhibit temperature dependent sol–gel transitions in the temperature range of 60–80 °C [32]. The molecular dynamic simulation study including solvent accessible surface area, number of hydrogen bonds, radial distribution functions, and the interaction energies at three different temperatures (25, 50, and 75 °C) has indicated that the increase in the temperature is accompanied by a decrease in the interactions between methylcellulose chains and water molecules and the increase in the interactions between methylcellulose chains, which has led to the thermo-responsive hydrogel formation by methylcellulose chains [33]. Several chemical modifications or physical mixing with other biopolymers were applied to attain sol–gel transition near body temperature [34]. The thermo-responsive hydrogel system made of methylcellulose and hyaluronic acid was applied for mucosal healing and the macromolecular drug (BSA) loaded hydrogel showed sol–gel transition near body temperature [35]. The BSA release was 50.0% of the loaded drug within 2 h and the system showed acceptable toxicity towards intestinal (colon) Caco-2 epithelial cells, even at high concentrations [35]. The synergistic hydrogel system made of methylcellulose and alginate showed superior thermo-responsiveness and viscosity than the individual components, and the gel system was used to load epidermal growth factors for skin regeneration [36]. The gel system is reported to provide the loaded EGF with thermal protection in a topical delivery format due to its promising rheological tenability and the system offered biocompatibility [36]. The formulation from methylcellulose and alginate showed in situ gel formation at 37 °C and was used as drug delivery systems for acetaminophen, mexiletine, metoprolol, ambroxol, loxoprofen, theophylline, ketotifen, and salbutamol [37]. The thermo-responsive hydrogel drug delivery system made of 1.0% (*w/v*) methylcellulose in water showed sol–gel transition at 60 °C and was applied for the delivery of ophthalmic drugs [38]. The use of different salts decreased the gelling temperature of methylcellulose formulation to near physiological temperature and increased the ocular bioavailability of the drug [38]. The injectable methylcellulose-based hydrogel system formulated in combination with collagen and beta glycerophosphate showed sol–gel transition at ~36 °C, and the gel system was found to be sufficiently robust to resist significant disintegration in the presence of phosphate-buffered saline [39]. The hydrogel system was reported to be a clinically translatable delivery system for stem cells and therapeutic molecules in vivo. It maintained the viability of human mesenchymal stem cells (hMSCs) encapsulated within it and confirmed cell proliferation by showing raised levels of dsDNA at increasing time points [39]. The thermo-responsive hydrogel developed from methylcellulose and kappa carrageenan was applied as drug delivery system [40]. The thermal transitions (sol–gel and gel–sol) of methylcellulose/water, kappa carrageenan/water, and methylcellulose/kappa carrageenan/water mixtures were investigated via differential scanning calorimetry, oscillatory rheological method, and small-angle X-ray scattering in the temperature range of 20–80 °C, and the mixed formulation of methylcellulose/kappa carrageenan/water showed a double thermal transition (gel–sol–gel) upon heating where there was a liquid state of the formulation between the low-temperature and high-temperature gel-state [40]. The injectable thermo-responsive hydrogel system made of methylcellulose and chitosan was used as three-dimensional synthetic matrix for tissue engineering and bone substitutes [41]. The bio-based blend in the presence of some salts (NaCl, Na_3_PO_4_, NaHCO_3_, and glycerophosphate) showed temperature-dependent gel formation at 37 °C, and the nature of salt influenced the gelation temperature and rate of the bio-based blend [41]. Thermo-responsive hydrogels made of methylcellulose were applied as promising substrates for cell-sheet engineering, and the addition of two saline solutions (Na_2_SO_4_ and phosphate buffered saline) lowered the LCST of the hydrogels made of 8% (*w*/*v*) methylcellulose [42].

Xyloglucan, found in the primary cell walls of many higher plants is made up of *β*-1, four linked glucan units substituted with xylose (Figure 3). Depending on the source of xyloglucan, the xylose units are further substituted with the galactose, and the sol–gel transition temperature of xyloglucan formulations was found to be reduced with the increase in the galactose removal ratio [43]. The xyloglucan derived from tamarind seed was treated with *β*-galactosidase to form partially de-galactosylated xyloglucan (45% of galactose residues removed), which was further used to develop thermo-responsive hydrogel with sol–gel transition at 27 °C [44]. The ex-vivo permeation of drugs from the gel was reported to be sustained, and in vitro drug release followed Higuchi rate model for first 5 h via an anomalous transport mechanism. A histological study showed that the administration of xyloglucan gel did not show any damage to nasal mucosa [44]. The bioinspired thermo-responsive hydrogels composed of xyloglucan and cellulose nanocrystals showed a reversible thermal transition at 35 °C, and exhibited tunable mechanical performance or changes in volume that made them suitable for biomedical applications including drug delivery, wound healing dressings, and implants [45]. The thermo-responsive property of xyloglucan was obtained by enzymatic de-galactosylation of tamarind seed xyloglucan, which reduced the galactose residue content by ∼50%, and the adsorption behavior indicated that de-galactosylated xyloglucan formed a more rigid layer on cellulose nanocrystals than that of native xyloglucan [45]. The de-galactosylated xyloglucan was applied to develop thermo-responsive hydrogel for the intraperitoneal administration of mitomycin C and in vitro drug release from the hydrogel following the Higuchi rate model over the time period of 5 h [46]. The intraperitoneal administration of mitomycin C in the loaded form produced a broad concentration time profile in both ascites and the plasma over a period of 3 h, while the drug in the solution form showed rapid disappearance from both sites [46]. The in situ thermo-responsive hydrogel of de-galactosylated xyloglucan was used for the oral administration of indomethacin and diltiazem, and the bioavailability of drugs from the xyloglucan gels was higher than that from control suspension [47]. The partially de-galactosylated xyloglucan at a concentration of 1.0 and 1.5 weight (%) showed sol–gel transition at 37 °C and followed the Higuchi rate model over a period of 5 h at a pH of 6.8 [47]. The ocular delivery of pilocarpine by in situ thermo-responsive hydrogel of partially de-galactosylated xyloglucan showed the sustained release of the drug, and the duration of miotic response was increased with increase in xyloglucan concentration [48]. The formulations made of enzymatically degraded xyloglucan (1.0, 1.5, and 2.0 weight%) showed sol–gel transition at 37 °C and followed the Higuchi rate model over a period of 6 h [48]. The in situ gelling system made of modified tamarind seed xyloglucan was applied for direct nose-to-brain delivery of an anti-epileptic drug, rufinamide [49]. The formulation showed gelation below 35 °C, and the results of pharmacokinetic studies in rats for direct nose-to-brain uptake of rufinamide demonstrated the superiority of the nasal in situ gel formulation over oral formulation of rufinamide [49]. The hydrogel made of xyloglucan was applied as a scaffold for tissue engineering which is useful for repairing damaged neural pathways in the central nervous system [50]. The bioengineered composite scaffolds developed from embedding electrospun poly(l-lactic acid) short nanofibers into a thermo-responsive xyloglucan hydrogel were easily injected into the injured brain, and the glial-derived neurotrophic factor was covalently attached onto and/or blended into the composite scaffolds to promote cell survival and axonal growth [51]. The bioengineered scaffolds showed sustained delivery of GDNF in vitro and confirmed the ability to support ventral midbrain (VM) dopamine progenitors [51].

Chitosan, made of *β*-1, 4 linked glucosamine units, can be obtained from the exoskeleton of shrimp and lobster after alkaline deacetylation of chitin (Figure 3). The thermo-responsive hydrogel systems were developed from chitosan of various molecular weights and applied for intranasal delivery of ibuprofen [52]. The thermosensitive nasal formulations showed in situ gelation with desirable spray characteristics at room temperature, and the muco-adhesive behavior of the semi-solid at physiological temperature due to phase change rendered potential to efficiently deliver therapeutics to brain [52]. The physically cross-linked injectable thermo-responsive hydrogel of chitosan was developed for the effective and sustained delivery of disulfiram to the cancer cells and these gel systems showed excellent biocompatibility and cytotoxicity in a dose-dependent manner on SMMC-7721 cells [53]. The formulation injected at room temperature followed rapid gel formation at body temperature and showed stronger cellular uptake than free disulfiram [53]. The thermo-responsive hydrogel developed with chitosan and *α* (alpha) *β*-glycerophosphate showed sol–gel transition at 37 °C and was applied as drug delivery system for the sustained release of adriamycin and 6-mercaptopurine [54]. The release rate of drugs from the hydrogel was found to be decreased with increase in the molecular weight of chitosan [54]. The thermo-responsive gel system of chitosan and *β*-glycerophosphate combined with desferrioxamine and human mesenchymal stem cells was applied as an injectable, multimodal, pro-angiogenic therapeutic for the treatment of critical limb ischaemia, and the gel system showed a sustained and biologically active release of desferrioxamine over the space of seven days [55]. This formulation showed a sol–gel transition at 33 °C, and the vascular endothelial growth factor (VEGF) expression in gel-exposed human umbilical vein endothelial cells was increased by the loaded components within the gel that resulted in a synergistic enhancement in the bioactivity [55]. The thermosensitive hydrogel designed by simple mixing of non-toxic quaternized chitosan (*N*-[(2-hydroxy-3-trimethylammonium) propyl] chitosan chloride), *αβ*-glycerophosphate, and poly(ethylene glycol) showed sol–gel transition at 37 °C and was applied for the nasal delivery of insulin [56]. The formulation did not show cytotoxicity for the nasal epithelial cells of mice [56]. The gelatin was added to the thermo-responsive hydrogel system of chitosan/*β*-glycerol phosphate to develop collagenase carrier for tendon bone healing in rabbit model and injection of the formulation with collagenase improved the healing of tendon-to-bone in rabbits [57]. A thermo-responsive hydrogel system composed of *N*-trimethyl chitosan chloride and *β*-glycerophosphate showed sol–gel transition at around 37 °C and was applied as a drug delivery system [58]. Tissue engineering applications of chitosan-based thermo-responsive hydrogels were shown in a minimally invasive manner, and the formulation made of chitosan and glycerol phosphate combined with encapsulated adipose-derived stromal cells was applied as an injectable scaffold for articular cartilage regeneration [59]. The addition of starch in the formulation did not change the sol–gel transition temperature but made the hydrogel network mechanically stronger as obtained by rheological study. The cytotoxicity screening in vitro proved that all materials used in the formulation were biocompatible [59]. The poly-D-lysine-functionalized thermo-responsive hydrogel made of chitosan and glycerophosphate salt was applied for neural tissue engineering and the immobilization of poly-D-lysine onto chitosan via azidoaniline photo-coupling improved cell adhesion and neurite outgrowth [60]. The thermo-responsive hydrogel system was reported to be capable of providing a suitable 3D scaffolding environment for neural tissue engineering [60].

The sol–gel transition type and biomedical applications of polysaccharide-based thermo-responsive hydrogels are summarized in Table 1. The polysaccharide based hydrogels accept easy chemical modifications on their backbones, and the physical mixing with other biopolymers or small synthetic molecules enhances their in situ gel forming capability without modifying their intrinsic biodegradability, biocompatibility, and non-toxicity. Nevertheless, polysaccharide-based thermo-responsive formulations often face some practical challenges including the weak mechanical strength of hydrogels and also fail to show sol–gel transition near body temperature.

## 3. Thermo-Responsive Proteins/Polypeptides and Their Drug Delivery Applications

Some proteins such as gelatin, collagen, and elastin-like polypeptides can show thermo-responsive properties, and the chemical modification or physical blending with other compounds can render them more suitability and sustainability towards drug delivery and tissue engineering applications [61].

**Figure 5:**
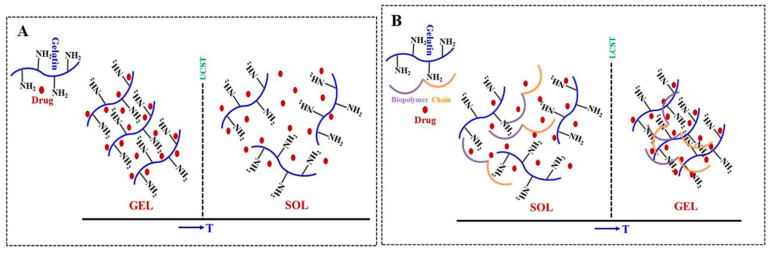
(**A**) UCST-type sol–gel transition of drug-loaded hydrogel system of gelatin; and (**B**) LCST-type sol–gel transition of drug-loaded hydrogel system of physically blended gelatin and other biopolymer (Schematic presentation).

Gelatin is obtained from boiling animal tissues such as beef bones, cartilage, tendons, and pig skin after acidic/alkaline hydrolysis or enzymatic/thermal degradation of collagen, and contains a three-helix structure with repeating sequences of glycine, proline, and alanine. Gelatin is a UCST-type thermo-responsive polymer (Figure 5A), and above a UCST of 40 °C, gelatin can be dissolved in an aqueous medium by forming random single coils which turn into triple helical structure upon cooling by hydrogen bonding and van der Waals force [30,62]. Thereby, gelatin was reported to be combined with other polymers to attain suitable gelling properties and ensure stability under body temperature conditions (Figure 5B), and those modifications should be useful for tissue engineering and drug delivery applications. The chitosan-gelatin thermo-responsive hydrogel was applied as a dual drug delivery system of curcumin-loaded nanoparticles and latanoprost for glaucoma treatment, and the eye drop formulation for dual drug delivery showed both in vitro and in vivo biocompatibility [63]. The oxidative stress-mediated damage in trabecular meshwork cells was effectively decreased by the treatment with this hydrogel containing curcumin-loaded nanoparticles via the reduction in inflammation-related gene expression, mitochondrial reactive oxygen stress (ROS) production, and the decrease in the apoptosis levels [63]. The thermosensitive hydrogel system based on chitosan and gelatin was applied as an injectable drug delivery system for the treatment of osteoarthritis by minimal invasive surgery, and the glutathione loaded into the formulation showed sustained release of glutathione that decreased reactive oxygen species level in Cisd2-deficient chondrocytes [64]. The injectable hydrogel system made of gelatin, chitosan, and *β*-glycerol phosphate was applied as cell carrier for nucleus pulposus regeneration and used in minimal invasive intervertebral disc surgery [65]. The hydrogel system showed sol–gel transition at body temperature and did not show cytotoxicity on nucleus pulposus cells [65]. The thermo-responsive hydrogel made of gelatin, chitosan, and *β*-glycerol phosphate disodium salt was applied as collagenase carrier in the tendon–bone junction to prolong the healing process within the tendon–bone interface, and histological analyses showed early healing and more bone formation at the tendon–bone interface after partial digestion of collagenase [57]. The erythropoietin-loaded injectable thermo-responsive hydrogel of chitosan and gelatin was applied to effectively enhance maxillary sinus floor augmentation in vivo, which is normally used for new bone formation before implant placement [66]. The thermo-responsive biodegradable hydrogel scaffold developed using gelatin and glycidyl methacrylated dextran was loaded with bone morphogenetic proteins in microspheres, and showed sustained release of the protein [67]. The thermomechanical scaffold with macroporous structure (6.0–38.0 micron) was generated by radical cross-linking and low dose γ-radiation [67]. The thermo-responsive biocompatible hydrogel of chitosan and gelatin showing sol–gel transition at physiological temperature was used as a bio-printing ink, which showed significant potential to print 3D, sterile, and cell-laden structures without post-processing using an inexpensive bio-printer [68].

**Figure 6:**
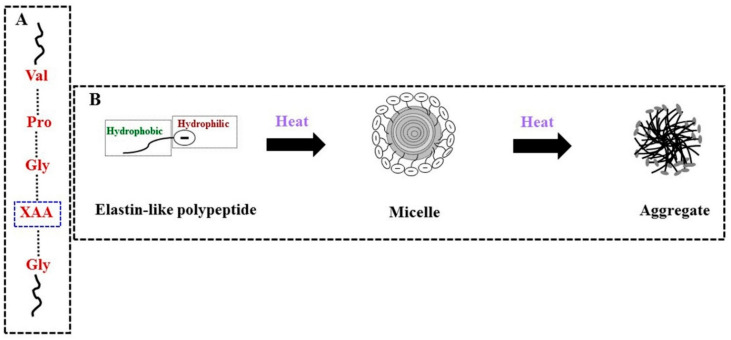
(**A**) The consensus the repeat sequence of elastin-like polypeptide (XAA: any amino acid except proline); and (**B**) Sol–gel transformation of elastin-like polypeptide with an increase in temperature. The single molecule of the elastin-like polypeptide is made of hydrophilic and hydrophobic blocks and starts to form aggregates upon heating through micellar intermediate formation. Above inverse transition temperature of both hydrophobic and hydrophilic blocks, elastin-like polypeptides start to form aggregates. In addition, upon cooling below their inverse transition temperature, the aggregate changes to single molecules in the solution.

Elastin-like polypeptides are biosynthesized by genetic engineering using elastin-like recombinamers [69,70]. Elastin-like polypeptides bear multiple copies of the consensus repeat (penta-peptide of valine-proline-glycine-any amino acid (except proline)-glycine) of native elastin (Figure 6A), and this sequence is known to be responsible for the LCST-type behavior of elastin-like polypeptides [69]. The thermo-responsive behavior of elastin-like polypeptides is shown in Figure 6B. The thermo-responsive behavior of elastin-like polypeptides depends on its molecular weight and concentration in the solution and the composition of the amino acids. Drug delivery systems based on elastin-like polypeptides are currently being applied synergistically with hyperthermia technology, and these drug delivery systems increase the efficacy of drug targeting and minimize the side effects of administration especially for cancer treatment. The thermo-responsive nano-gel based on elastin-b-collagen-like peptide was used for sustained delivery of drug into collagen-rich matrices [71]. The temperature sensitivity of the nano-gel was developed from tethered elastin domain of elastin-b-collagen-like peptide, and the cell viability and proliferation studies using fibroblasts and chondrocytes indicated that nano-gel was highly biocompatible [71]. The promising method involving elastin-like polypeptides is being applied in cancer therapy and anticancer drugs such as doxorubicin or paclitaxel are conjugated to the polypeptides for the targeted delivery of the drug to the tumor sites [72]. The thermo-responsive protein hydrogel was developed by fusing the LCST-type elastin-like polypeptides with elastomeric protein made of tandemly arranged globular proteins in the folded form, and the engineered protein polymers based on fusion proteins showed better aqueous solubility and temperature-dependent micellization [73]. The hydrogel based on the fusion proteins are reported to have versatile biomedical applications including drug and cell delivery, tissue engineering, and other biological applications [73]. The biodegradable and biocompatible amphiphilic block copolymers based on polysaccharide and stimuli-responsive elastin-like polypeptide are fabricated via copper(I)-catalyzed azide-alkyne cycloaddition, and both blocks can form well-defined nanoparticles via thermo-responsive self-assembly in aqueous media above a suitable transition temperature [74]. Thereby, the promising bio-inspired drug delivery systems based on elastin-like polypeptide are found to be suitable for various potential biomedical applications.

The sol–gel transition type and biomedical applications of protein/polypeptide-based thermo-responsive hydrogels are summarized in Table 2. The drug delivery systems based on thermo-responsive proteins/polypeptides can show tunable properties. Nevertheless, the numbers of natural proteins with LCST-type thermo-responsive properties are very few. Therefore, the search for protein-based thermo-responsive drug delivery systems having LCST-type thermo-responsive property and enough mechanical integrity is underway.

## 4. Future Prospects of Thermo-Responsive Biopolymers

Textile-based transdermal therapy is a process in which drug can be delivered to the affected sites on skin by non-invasive manner with controlled and sustained drug release properties [75,76]. Bio-functional textiles used in transdermal delivery of drug are developed combining textile fabrics with drug carriers, and these materials are used to make progress toward a new generation of wearable drug delivery devices [77]. It does not require the use of needles for administration of the drug, and the bioavailability of the drug can be enhanced effectively by this method [78]. The double layer fabric with an outer layer of polypropylene (15%) and an inner layer of nylon (72.5%) with polyurethane (12.5%) showed better transdermal delivery of rhodamine B base and caffeine than control clothing made of pure organic cotton [79]. The modification of textile fabrics with thermo-responsive hydrogels as drug carriers is a recent approach to develop bio-functional textiles, and these materials can improve the condition of patients with atopic dermatitis through textile-based transdermal therapy, which offers the sustained and timely delivery of drug molecules to the affected areas on the skin [80]. The thermo-responsive micro-hydrogel made of poly(*N*-isopropylacrylamide) was developed from an inverse suspension polymerization method, and the procaine-loaded micro-hydrogel system was used for the designing of bio-functional textiles [81]. The transdermal experiments at different temperatures indicated that the drug carriers on the bio-functional textiles could give sustained and controlled release of procaine [81]. Our research team had developed some LCST-type PF127-based drug delivery systems to treat skin diseases such as atopic dermatitis via textile-based transdermal therapy [16,25,28,29]. PF127-based stimuli-responsive hydrogels showed transdermal delivery of Cortex Moutan and exhibited sol–gel transitions around 37 °C [29]. The thermo-responsive hydrogels made of PF127 and carboxymethyl cellulose sodium worked as a drug delivery system of traditional Chinese medicine, Cortex Moutan and supplied moisture to the skin as well [25]. The PF127-based hydrogel system loaded with a Chinese herbal drug was a suitable candidate for transdermal therapy after being coated onto a cotton fabric [25]. In spite of PF127-based hydrogel systems loaded with gallic acid were found to be minimally cytotoxic on skin cells in a recent scientific report [28]. In this respect, transdermal drug delivery systems based on thermo-responsive biopolymers could be a better alternative for sustained delivery of drug [30]. The therapeutic system developed from Metolose (methylcellulose and hydroxypropyl methylcellulose) can exhibit thermo-responsive properties, and was applied as transdermal drug delivery systems [82]. The clothes using bioactive textiles, which were developed by coating cotton fabric or chemically modified cotton (having aldehyde or carboxymethyl functional groups) with chitosan materials (crosslinking with glutaraldehyde or natrium tripolyphosphate) loaded with biologically active substances extracted from plants (Viola Tricolor), were used for delivering plant extracts with antioxidant activity to people with allergies or other skin problems via textile-based transdermal therapy [83]. The physical mixing of thermo-responsive biopolymers with some natural polymers with pH-responsive properties such as alginate, chitosan, hyaluronic acid, and pectin can provide the bio-based formulations with the ability to swell or shrink in response to pH of the external media, and depending on the pH of external media, pH-responsive polymers can self-assemble to form hydrogels with controlled and sustained drug releasing properties [84]. The dual-responsive hydrogels, which can simultaneously show sensitivity towards changes in temperature and pH, are being designed from thermo- and pH-responsive biopolymers, respectively, for potential drug delivery applications [85]. Both temperature and pH are critical in biological systems, and drug delivery systems based on dual-responsive hydrogels can provide precision in the controlled and site-specific delivery of drugs as it is more adaptable to the complex environment of human body fluid [86].

## 5. Conclusions

The hydrogel systems based on thermo-responsive polymers such as PF127 find multi-directional drug delivery and tissue engineering applications such as pluronics can show thermo-gelation at physiological temperatures, and drug delivery systems are reported to have advanced properties such as site-specific delivery along with controlled and sustained delivery of drug molecules. Our group has published some articles on the transdermal delivery of drugs for the treatment of atopic dermatitis using PF127-based hydrogels. Nevertheless, PF127-based hydrogel systems were reported to be slightly cytotoxic by some research groups and the degradation products of those gel system were not clearly mentioned. In this review, the main focus has been given to thermo-responsive biopolymers including carbohydrates, proteins, and polypeptides, which are biodegradable, biocompatible, and, mostly, non-cytotoxic. The manuscript has described the chemical nature, rheological properties, and drug delivery applications of several LCST-type formulations made of natural polymers: cellulose, dextran, chitosan, xyloglucan, and gelatin- and elastin-like polypeptides. Drug delivery systems were developed by the physical mixing of thermo-responsive biopolymers with other natural polymers in order to modulate their temperature sensitivity to near body temperatures and increase their suitability and sustainability as drug carriers. Bio-functional textiles developed by applying stimuli-responsive hydrogel-based drug delivery systems of PF127 on textile fabrics were reported to be capable of delivering both drug and moisture to the affected area of eczema (atopic dermatitis) patients. The future perspective for textile-based transdermal drug delivery systems based on thermo-responsive natural polymers is discussed to develop an invasive treatment method for atopic dermatitis.

## Figures and Tables

**Table 1:** The sol–gel transition type and biomedical applications of thermo-responsive polysaccharide-based hydrogels.

Polysaccharide	Properties	Structural Units (Monosaccharides)	Origin/Source (Natural)	Sol–Gel Transition Type	Biomedical Applications	
					Drug Delivery [Ref]	Tissue Engineering [Ref]
Cellulose (Methyl Cellulose)	(i) biodegradable, nontoxic, and biocompatible (ii) hydrogel strength is high	*O*-methylated D-glucopyranose and D-glucopyranose units	Green plants and many varieties of algae	LCST	[35,36,37,39]	[41,42]
Xyloglucan	(i) biodegradable, injectable, nontoxic, and biocompatible (ii) hydrogel strength is average	glucan units substituted with xylose	Primary cell wall of many higher plants	LCST	[44,45,48,49]	[50,51]
Chitosan	(i) biodegradable, nontoxic, and biocompatible (ii) hydrogel strength is medium or low	D-glucosamine and *N*-acetyl-D-glucosamine	Exoskeleton of crustaceans	LCST	[52,53,55,57]	[59,60]

**Table 2:** The sol–gel transition type and biomedical applications of thermo-responsive protein/polypeptide-based hydrogels.

Protein/Polypeptide	Properties	Structural Units (Monosaccharides)	Origin/Source (Natural)	Sol–Gel Transition Type	Biomedical Applications	
					Drug Delivery [Ref]	Tissue Engineering [Ref]
Gelatin (protein)	(i) biodegradable, injectable, nontoxic, and biocompatible (ii) hydrogel strength is poor	Glycine, proline, alanine, and other amino acids	Animal tissues such as beef bones, cartilage, tendons, and pig skin	LCST *^A^*	[63,64,65]	[66,67,68]
Elastin-like polypeptides	(i) biodegradable and biocompatible (ii) mechanical strength is poor	Valine, proline, glycine, and other amino acids	Human tropoelastin	LCST	[72,73]	[70,74]

*^A^* Gelatin physically blended with other biopolymers.

## Data Availability

The data presented in this study are available on request from the corresponding author.
